# Lower SHBG level is associated with higher leptin and lower adiponectin levels as well as metabolic syndrome, independent of testosterone

**DOI:** 10.1038/s41598-017-03078-0

**Published:** 2017-06-02

**Authors:** Chia-Chu Liu, Shu-Pin Huang, Kai-Hung Cheng, Tusty-Jiuan Hsieh, Chun-Nung Huang, Chii-Jye Wang, Hsin-Chih Yeh, Chia-Chun Tsai, Bo-Ying Bao, Wen-Jeng Wu, Yung-Chin Lee

**Affiliations:** 1 Department of Urology, Kaohsiung Medical University Hospital, Kaohsiung Medical University, Kaohsiung, 807 Taiwan; 2 Department of Urology, Faculty of Medicine, College of Medicine, Kaohsiung Medical University, Kaohsiung, 807 Taiwan; 3 Department of Urology, Pingtung Hospital, Ministry of Health and Welfare, Pingtung, 900 Taiwan; 4 Division of Cardiology, Department of Internal Medicine, Kaohsiung Medical University Hospital, Kaohsiung, 807 Taiwan; 5 Graduate Institute of Medicine, College of Medicine, Kaohsiung Medical University, Kaohsiung City, 807 Taiwan; 6 Research Center for Environmental Medicine, Kaohsiung Medical University, Kaohsiung, 807 Taiwan; 7 Department of Urology, Kaohsiung Municipal Ta-Tung Hospital, Kaohsiung, 801 Taiwan; 8 Department of Pharmacy, China Medical University, Taichung, 404 Taiwan

## Abstract

In addition to testosterone (T), the emerging role of sex hormone-binding globulin (SHBG) in pathogenesis of metabolic syndrome (MetS) has been noted recently. However, reports of associations with serum adipocytokine levels are still limited. Therefore, we conducted this study to evaluate whether serum T and SHBG levels are independent predictors for the risk of MetS that are associated with adiponectin and leptin levels in 614 Taiwanese men over 40 years old collected from a free health screening. Subjects in the lowest quartile of TT and SHBG levels are exposed to a 1.58 and 3.22 times risk of developing MetS, as compared to those in the highest quartile of TT and SHBG levels. However, SHBG retains its significance independent of TT as a MetS risk predictor, but not vice versa. In addition, SHBG was significantly correlated with both adiponectin and leptin levels even after adjusting for TT levels. In conclusion, SHBG served as a major predictor for the risk of MetS and was correlated with serum adiponectin and leptin levels that are independent of T. Further studies are needed to elucidate the true role of SHBG in the pathogenesis of MetS and possible mechanisms associated with serum adiponectin and leptin levels.

## Introduction

Metabolic syndrome (MetS) is a collection of cardio-metabolic risk factors, including obesity, insulin resistance, hypertension, and dyslipidemia, appearing to affect approximately 10–40% of adult populations worldwide^1^. It has become a major public health concern in the twenty-first century due to its rapidly increasing prevalence in recent years and its utility as a predictor for the risk of type 2 diabetes mellitus (DM) and cardiovascular disease^2, 3^. Patients with MetS have a 5-fold increase in risk for type 2 DM and twofold increase in risk for cardiovascular disease over the subsequent 5 to 10 years^3^. Increased adipose tissue with dysregulation of adipocytokines is thought to play a major role in the pathogenesis of MetS^4^. Adiponectin and leptin are the two most important adipocytokines that can modulate insulin sensitivity, body weight and inflammatory status^5^.

In East Asia, the prevalence of MetS has been reported higher in men and increased with age^6–8^. Testosterone (T) is the principal sex hormone in men with both androgenic and anabolic effects that decline gradually, especially after the age of 40^9^. It consists of three components, including T bound to sex hormone‑binding globulin (SHBG), T bound to albumin, and free testosterone (FT)^10, 11^. Traditionally, FT is considered the main component with hormonal activity, and serum SHBG is considered a transport protein for T to target tissues, regulating the circulating concentration of FT^10, 11^. However, recent research has demonstrated that serum SHBG can directly affect cells by binding to their receptors and acting as a hormone^12^. In addition, polymorphisms in the SHBG gene have been associated with the risk of type 2 DM, suggesting a causal role for SHBG in metabolic disease risk^13, 14^. A large number of epidemiological studies have also reported that both low T and SHBG levels can increase the risk of MetS in men^15^. Some studies even suggested that serum SHBG levels may play a more dominant role than T levels in determining the risk of MetS^16–18^.

Although many studies have investigated the relationships of serum T and SHBG levels with the risk of MetS in men, only a few have focused on their relationships with serum adipocytokines, especially adiponectin and leptin levels. Therefore, we conducted this study to evaluate whether serum T and SHBG levels are independent predictors for the risk of MetS that are associated with adiponectin and leptin levels in a large sample of middle- to old-aged Taiwanese men.

## Results

Of the 656 men participating in the health screening, 42 subjects were excluded due to current malignancies (13 cases), current use of medications that would interfere with the measurement of natural testosterone levels (17 cases) and incomplete evaluation (12 cases), leaving 614 subjects with a mean age of 55.8 ± 5.7 years (range: 43–83 years). The baseline characteristics of the study population are summarized in Table 1. The mean value of TT and SHBG levels were 390.6 ± 97.1 ng/dl (median: 384.5, interquartile range (IQR): 317.8–458.3) and 43.0 ± 19.4 nmol/L (median: 39.5, IQR: 29.4–50.9) respectively. 234(38.1%) participants were diagnosed as having MetS.

**Table 1:** Baseline characteristics of study population (N = 614).

	N (%)	Mean ± SD	Range
Age (year)		55.8 ± 5.7	43–83
BMI (kg/m^2^)		25.6 ± 6.2	18–37.5
Waist circumference (cm)		86.4 ± 7.0	66–111
Systolic blood pressure (mmHg)		131.2 ± 12.1	93–176
Diastolic blood pressure (mmHg)		82.6 ± 8.6	58–113
Education status			
At least some college	362 (59.0)		
Secondary/high school	232 (37.8)		
Primary school or less	20 (3.2)		
Hypertension	180 (29.3)		
Diabetes	65 (10.6)		
Current smoking	83 (13.5)		
Current alcohol drinking	96 (15.6)		
Regular exercise	380 (61.9)		
Metabolic syndrome	234 (38.1)		
Laboratory Data			
Total testosterone (ng/dl)		390.6 ± 97.1	101.0–740.0
Free testosterone (ng/dl)		6.92 ± 2.02	1.82–13.90
SHBG (nmol/L)		43.0 ± 19.4	6.9–145.0
Albumin (g/dl)		4.46 ± 0.22	3.70–5.20
Fasting blood glucose (mg/dl)		100.1 ± 20.9	68.0–219.0
Triglyceride (mg/dl)		133.5 ± 91.8	31.0–1396.0
HDL (mg/dl)		47.5 ± 10.7	25.7–91.3
Adiponectin (μg/ml)		11.6 ± 6.7	0–53.9
Leptin (ng/ml)		4.13 ± 2.54	0.63–18.03

Abbreviation: BMI = body mass index; SHBG = sex hormone binding globulin; HDL = high density lipoprotein.

### Clinical characteristics and laboratory data in subjects with and without MetS

**Figure 1:**
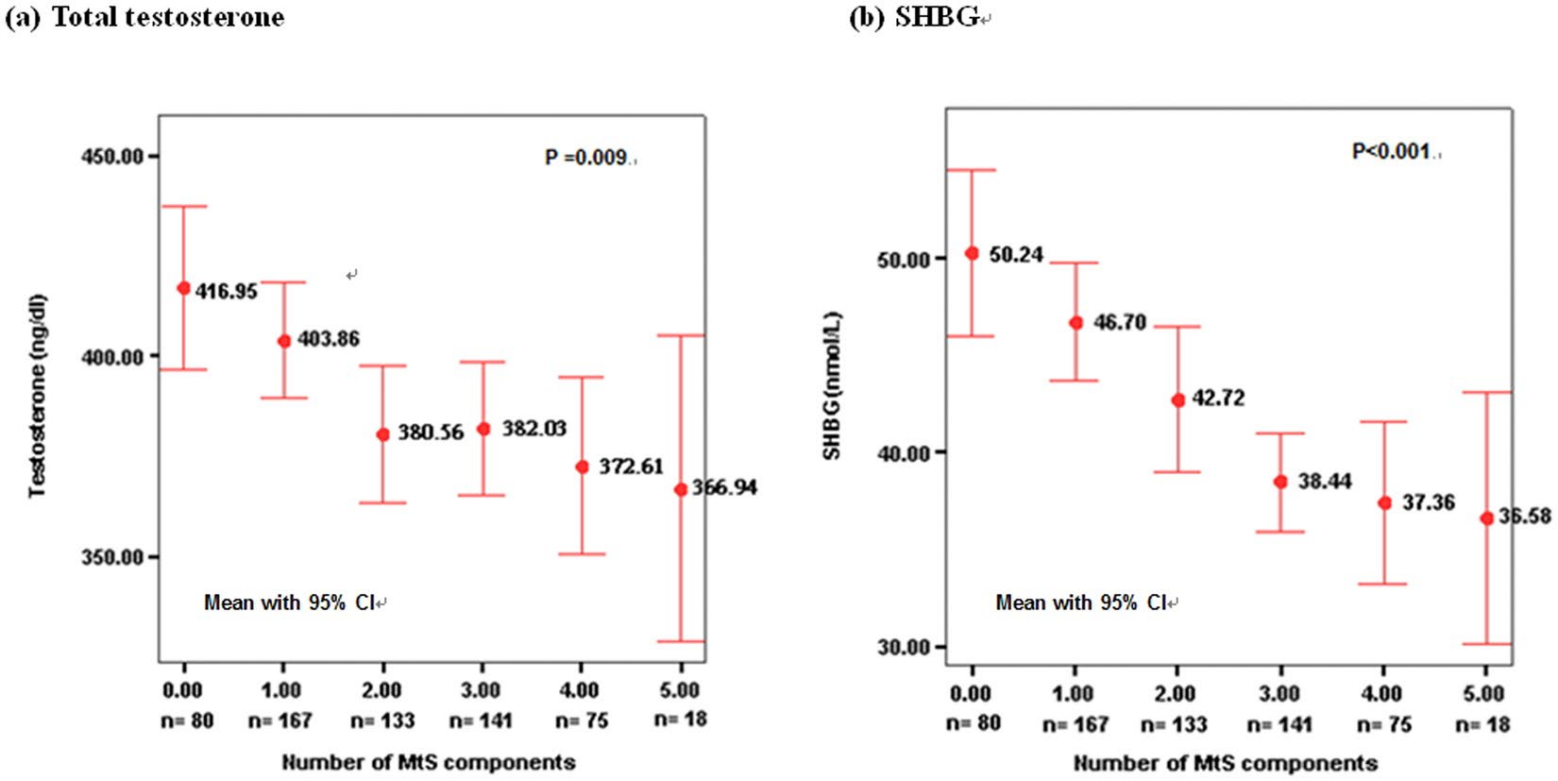
Distribution of total testosterone and sex hormone binding globulin (SHBG) levels according to the number of metabolic syndrome (MetS) components.

Table 2 compares the clinical characteristics and laboratory data in subjects with and without MetS. Subjects with MetS had significantly greater age (56.9 ± 6.0 vs 55.0 ± 3.6 years, p < 0.001), higher BMI (27.6 ± 9.2 vs 24.4 ± 2.2 kg/m^2^, p < 0.001), lower education levels (at least some college: 52.1% vs 63.2%, p = 0.001), higher prevalence of current smoking (18.5% vs 10.5%, p = 0.009) and alcohol drinking (19.3% vs 13.5%, p = 0.014), lower serum TT (377.9 ± 97.1 vs 398.5 ± 96.4 ng/dl, p = 0.011), SHBG (38.0 ± 16.0 vs 46.1 ± 20.6 nmol/L, p < 0.001) and adiponectin (7.8 ± 4.5 vs 13.9 ± 6.8 μg/ml, p < 0.001) levels, and higher leptin levels (5.47 ± 2.90 vs 3.32 ± 1.89 ng/ml, p < 0.001) than those without MetS (Table 2). No significant difference in FT level was noted between subjects with and without MetS. Figure 1 shows the mean concentrations of TT and SHBG according to the number of MetS components. Both TT and SHBG concentrations decreased gradually with increasing number of MetS components (p for trend: 0.009 and < 0.001, respectively).

**Table 2:** Comparison of clinical characteristics and laboratory data between. subjects with and without metabolic syndrome (MetS).

	Subjects with MetS (n = 234)	Subjects without MetS (n = 380)	P value
Age (year)	56.9 ± 6.0	55.0 ± 3.6	<0.001
BMI (kg/m^2^)	27.6 ± 9.2	24.4 ± 2.2	<0.001
Waist circumference (cm)	90.6 ± 7.0	83.7 ± 5.5	<0.001
Education status, n(%)			0.001
At least some college	122 (52.1)	240 (63.2)	
Secondary/high school	98 (41.9)	134 (35.3)	
Primary school or less	14 (6.0)	6 (1.6)	
Hypertension, n(%)	111 (47.6)	69 (18.2)	<0.001
Diabetes, n(%)	49 (22.2)	17 (4.5)	<0.001
Current smoking, n(%)	43 (18.5)	40 (10.5)	0.009
Current alcohol drinking, n(%)	45 (19.3)	51 (13.5)	0.014
Regular exercise, n(%)	138 (59.0)	242 (63.7)	0.243
Laboratory Data			
Total testosterone (ng/dl)	377.9 ± 97.1	398.5 ± 96.4	0.011
Free testosterone (ng/dl)	6.87 ± 2.16	7.00 ± 1.77	0.44
SHBG (nmol/L)	38.0 ± 16.0	46.1 ± 20.6	<0.001
Albumin (g/dl)	4.54 ± 0.24	4.41 ± 0.19	<0.001
Fasting blood glucose (mg/dl)	109.8 ± 25.5	94.1 ± 14.8	<0.001
Triglyceride (mg/dl)	170.9 ± 86.4	110.3 ± 87.3	<0.001
HDL (mg/dl)	42.2 ± 8.5	50.7 ± 10.6	<0.001
Adiponectin (μg/ml)	7.8 ± 4.5	13.9 ± 6.8	<0.001
Leptin (ng/ml)	5.47 ± 2.90	3.32 ± 1.89	<0.001

Abbreviation: MetS = metabolic syndrome; BMI = body mass index; SHBG = sex hormone binding globulin; HDL = high density lipoprotein

### Spearmen correlations of TT and SHBG with individual MetS components and adipocytokine levels

TT was significantly correlated with waist circumference (WC) (r = −0.128, p = 0.002), diastolic blood pressures (r = −0.088, p = 0.042), fasting blood glucose (FBG) (r = −0.094, p = 0.02) and triglyceride (TG) (r = −0.103, p = 0.011). SHBG was significantly correlated with WC (r = −0.245, p < 0.001), FBG (r = −0.115, p = 0.005), TG (r = −0.359, p < 0.001), and high density lipoprotein (HDL) (r = 0.244, p < 0.001) (Table 3). Both TT and SHBG were significantly correlated with adiponectin (r = 0.138, p = 0.001 and r = 0.315, p < 0.001, respectively) and leptin (r = −0.156, p < 0.001 and r = −0.240, p < 0.001, respectively) levels. Both adiponectin and leptin were significantly correlated with all individual MetS components (Table 3).

**Table 3:** Spearmans correlation of total testosterone and sexual hormone binding globulin (SHBG) levels with individual metabolic syndrome components and adipocytokine levels.

	Waist Circumference	Systolic BP	Diastolic BP	FBG	TG	HDL	Leptin	Adiponectin
**Total testosterone**	−0.128^**^	−0.064	−0.088^*^	−0.094^*^	−0.103^*^	0.066	−0.156^**^	0.138^**^
**SHBG**	−0.245^**^	−0.062	−0.055	−0.115^**^	−0.359^**^	0.244^**^	−0.240^**^	0.315^**^
**Adiponectin**	−0.341^**^	−0.145^**^	−0.146^**^	−0.287^**^	−0.419^**^	0.444^**^	−0.338^**^	1
**Leptin**	0.648^**^	0.158^**^	0.217^**^	0.234^**^	0.326^**^	−0.237^**^	1	

Abbreviation: SHBG = sex hormone binding globulin; BP = blood pressure; FBG = fasting blood glucose; TG = Triglyceride; HDL = high density lipoprotein.

^*^p < 0.05

^**^p < 0.01

### Relationships of TT and SHBG levels with the risk of MetS

In multivariate regression analyses, both TT and SHBG levels categorized by quartile were significantly negatively associated with the risk of MetS after adjusting for confounding factors of age, educational level, alcohol drinking, and cigarette smoking (p for trend = 0.021 and < 0.001, respectively). Subjects in the lowest quartile of TT and SHBG levels were exposed to 1.58 and 3.22 times the risk of having MetS respectively, when compared to those in the highest quartile of TT and SHBG levels (Table 4). When adjustment of both TT and SHBG levels were performed, only SHBG remained an independent predictor for the risk of MetS. Subjects in the lowest quartile of SHBG levels were exposed to 2.91 times the risk of having MetS when compared to those in the highest quartile of SHBG levels. The results were similar even after adjusting for BMI (Table 4). The sensitivity analysis to exclude subjects with DM demonstrated similar results (data not presented).

**Table 4:** Relationships of total testosterone and sexual hormone binding globulin (SHBG) levels with the risk of metabolic syndrome.

Total testosterone (ng/dl)	N	Mean ± SD	Adjusted OR (95% CI)		
			Model 1	Model 2^a^	Model 3
>75^th^	153	518.7 ± 54.6	Ref	Ref	Ref
50–75^th^	154	418.2 ± 20.3	0.83 (0.51–1.36)	0.64 (0.38–1.07)	0.61 (0.35–1.08)
25–50^th^	154	351.1 ± 19.5	1.26 (0.78–2.04)	0.89 (0.54–1.48)	0.78 (0.45–1.36)
<25^th^	153	273.2 ± 35.6	1.58 (0.98–2.55)	0.99 (0.59–1.66)	0.86 (0.48–1.52)
P for trend			0.021	0.67	0.86
**SHBG (nmol/L)**	**N**	**Mean ± SD**	**Model 1**	**Model 2** ^**b**^	**Model 3**
>75^th^	153	69.1 ± 18.6	Ref	Ref	Ref
50–75^th^	154	44.5 ± 3.4	1.44 (0.87–2.40)	1.40 (0.84–2.34)	1.35 (0.77–2.34)
25–50^th^	151	34.6 ± 3.0	1.62 (0.98–2.69)	1.52 (0.90–2.56)	1.53 (0.63–2.01)
<25^th^	156	24.0 ± 4.5	3.22 (1.96–5.29)	2.91 (1.71–4.95)	2.02 (1.12–3.62)
P for trend			<0.001	<0.001	0.038

Abbreviation: SD = standard deviation; OR = odds ratio; CI = confidence interval.

Model 1: adjusted for age, educational level, alcohol drinking and cigarette smoking.

Model 2^a^: Model 1 plus SHBG level.

Model 2^b^: Model 1 plus total testosterone level.

Model 3: Model 2 plus body mass index.

### Relationships of TT and SHBG levels with adiponectin and leptin levels

In multiple linear regression analyses, both TT and SHBG levels were significantly positively correlated with adiponectin levels (p = 0.002 and < 0.001, respectively) and negatively correlated with leptin levels (p < 0.001 and < 0.001, respectively) after adjusting for age, educational level, alcohol drinking, and cigarette smoking (Tables 5 and 6). However, when adjustment of TT and SHBG levels was performed, only SHBG was independently correlated with adiponectin levels (p < 0.001). Both TT and SHBG remained significantly correlated with leptin levels (p = 0.029 and < 0.001, respectively). The results were similar even after adjusting for BMI (Tables 5 and 6). The sensitivity analysis to exclude subjects with DM also demonstrated similar results (data not presented).

**Table 5:** Relationships of total testosterone with adiponectin and leptin levels.

Log_10_ Adiponectin	N	Mean ± SD	Model 1		Model 2		Model 3	
			ß (SE)	p-value	ß (SE)	p-value	ß (SE)	p-value
Total testosterone (ng/dl)	614	390.6 ± 97.1	0.125 (<0.001)	0.002	0.021 (<0.001)	0.613	0.017 (<0.001)	0.675
0–25^th^	153	273.2 ± 35.6	Ref	—	Ref	—	Ref	—
25–50^th^	154	351.1 ± 19.5	0.070 (0.028)	0.150	0.037 (0.027)	0.437	0.036 (0.027)	0.436
50–75^th^	154	418.2 ± 20.3	0.153 (0.028)	0.002	0.095 (0.028)	0.045	0.099 (0.027)	0.036
>75^th^	153	518.7 ± 54.6	0.135 (0.028)	0.006	0.016 (0.029)	0.748	0.014 (0.028)	0.781
P for trend				0.002		0.480		0.485
**Log**_**10**_ **Leptin**	**N**	**Mean ± SD**	**ß (SE)**	**p-value**	**ß (SE)**	**p-value**	**ß (SE)**	**p-value**
Total testosterone (ng/dl)	614	390.6 ± 97.1	−0.169 (<0.001)	<0.001	−0.114 (<0.001)	0.007	−0.105 (<0.001)	0.01
0–25^th^	153	273.2 ± 35.6	Ref	—	Ref	—	Ref	—
25–50^th^	154	351.1 ± 19.5	−0.025 (0.028)	0.617	−0.005 (0.028)	0.915	−0.005 (0.027)	0.920
50–75^th^	154	418.2 ± 20.3	−0.152 (0.028)	0.002	−0.119 (0.028)	0.016	−0.129 (0.027)	0.006
>75^th^	153	518.7 ± 54.6	−0.148 (0.028)	0.003	−0.080 (0.029)	0.124	−0.074 (0.028)	0.132
P for trend				<0.001		0.029		0.023

Abbreviation: SD = standard deviation; SE = standard error; OR = odds ratio; CI = confidence interval.

Model 1: adjusted for age, educational level, alcohol drinking, and cigarette smoking.

Model 2: Model 1 plus sexual hormone binding globulin level.

Model 3: Model 2 plus body mass index.

**Table 6:** Relationships of sexual hormone binding globulin (SHBG) with adiponectin and leptin levels

Log_10_ Adiponectin	N	Mean ± SD	Model 1		Model 2		Model 3	
			ß (SE)	p-value	ß (SE)	p-value	ß (SE)	p-value
SHBG (nmol/L)	614	43.0 ± 19.4	0.301 (0.001)	<0.001	0.294 (0.001)	<0.001	0.280 (0.001)	<0.001
0–25^th^	153	24.0 ± 4.5	1	—	1	—	1	—
25–50^th^	154	34.6 ± 3.0	0.096 (0.027)	0.039	0.094 (0.027)	0.044	0.084 (0.027)	0.073
50–75^th^	151	44.5 ± 3.4	0.257 (0.027)	<0.001	0.254 (0.028)	<0.001	0.237 (0.028)	<0.001
>75^th^	156	69.1 ± 18.6	0.343 (0.027)	<0.001	0.341 (0.029)	<0.001	0.321 (0.029)	<0.001
P for trend				<0.001		<0.001		<0.001
**Log**_**10**_ **Leptin**	**N**	**Mean ± SD**	**ß (SE)**	**p-value**	**ß (SE)**	**p-value**	**ß (SE)**	**p-value**
SHBG (nmol/L)	614	43.0 ± 19.4	−0.197 (0.001)	<0.001	−0.157 (0.001)	<0.001	−0.120 (0.001)	0.003
0–25^th^	153	24.0 ± 4.5	1	—	1	—	1	—
25–50^th^	154	34.6 ± 3.0	−0.098 (0.028)	0.041	−0.083 (0.028)	0.086	−0.054 (0.027)	0.242
50–75^th^	151	44.5 ± 3.4	−0.176 (0.028)	<0.001	−0.144 (0.029)	0.004	−0.097 (0.028)	0.045
>75^th^	156	69.1 ± 18.6	−0.273 (0.028)	<0.001	−0.232 (0.030)	<0.001	−0.178 (0.029)	<0.001
P for trend				<0.001		<0.001		<0.001

Abbreviation: SD = standard deviation; SE = standard error; OR = odds ratio; CI = confidence interval.

Model 1: adjusted for age, educational level, alcohol drinking, and cigarette smoking.

Model 2: Model 1 plus total testosterone level.

Model 3: Model 2 plus body mass index.

## Discussion

In a large sample of middle- to old-aged Taiwanese men, subjects with MetS had significantly lower serum TT and SHBG levels compared to those without MetS. As the number of MetS components increased, both serum TT and SHBG levels decreased gradually with significant trend. However, no significant difference of serum FT levels was noted in subjects with and without MetS. Subjects in the lowest quartile of TT and SHBG levels are exposed to 1.58 and 3.22 times higher risk of having MetS respectively, when compared to those in the highest quartile of TT and SHBG levels after adjusting for age and lifestyle factors (Table 4).

Recently, Brand and colleagues performed an updated meta-analysis from 20 observational studies and showed that men with low concentrations of TT, SHBG or FT were more likely to have prevalent MetS (odds ratios per quartile decrease were 1.69, 1.73 and 1.46 for TT, SHBG and FT, respectively) and incident MetS (odds ratios per quartile decrease were 1.25, 1.44 and 1.14 for TT, SHBG and FT, respectively) after adjusting for age and lifestyle factors^15^. According to their analysis, SHBG was the strongest and FT the weakest predictor for both prevalent and incident MetS^15^. In our study, both low TT and SHBG were significant predictors for the risk of prevalent MetS, but FT showed no significant association. Several cross-sectional studies also reported no significant difference in FT level between subjects with and without MetS^16, 19–21^. In the longitudinal studies of Kupelian *et al*.^22^ and Li *et al*.^23^, only TT and SHBG, but not FT were significant predictors for the risk of incident MetS in men. These results suggest that the impact of FT on MetS may be weak or confounded by several factors like discrepancy in the measurement of FT, definition of MetS and baseline characteristics of the different study populations^15, 16, 24^. Further studies are needed to clarify the real relationship between FT and MetS.

Although both TT and SHBG levels are significant predictors for the risk of MetS in our initial analyses, only SHBG retains its significance after adjustment of both TT and SHBG levels. Some studies have reported SHBG as a dominant predictor for prevalent and incident MetS, independent of TT, while TT lost its significance as a predictor for the risk of MetS after adjustment of SHBG^16, 17^. In our study, we also found that serum SHBG levels were significantly correlated with adiponectin and leptin levels even after controlling for age, lifestyle factors, BMI and TT level. Both adiponectin and leptin levels were significantly associated with all individual MetS components.

Adiponectin and leptin are two important cytokines secreted from adipose tissues that play important roles in the pathogenesis of MetS. Adiponectin has been found to have antidiabetic, anti-atherogenic, and anti-inflammatory properties, while leptin can regulate body weight by modulating appetite and energetic balance, and upregulate proinflammatory cytokines that are associated with insulin resistance and endothelial dysfunction^5, 25^. Aside from our study, only limited epidemiologic studies have evaluated the relationships among SHBG, adiponectin and leptin in men^26–28^. Gannage´-Yared *et al*. first reported that serum SHBG level was positively correlated with adiponectin level and negatively correlated with leptin level in men^26^. The relationship between SHBG and adiponectin persisted after adjustment for waist or BMI, while SHBG lost its relationship with leptin after adjustment for BMI^26^. Vanbillemont *et al*. used dual-energy X-ray absorptiometry to determine body composition and found that SHBG remained significantly associated with adiponectin after adjustment for body composition, while SHBG lost its association with leptin after adjustment for body composition^28^. In our study, we found that SHBG was significantly correlated with both adiponectin and leptin levels after adjusting for BMI in a large sample of middle- to old-aged Taiwanese men. The discrepancies reported in above epidemiologic studies might be owing to ethnic variations. The impact of race/ethnicity on the association of SHBG with adiponectin and leptin should be clarified by further studies. In addition, Simó *et al*. recently reported that adiponectin can increase hepatic SHBG production by upregulating hepatocyte nuclear factor 4 levels via changes in the hepatic lipid content in their *in vitro* study by using HepG2 cells^29^. Further studies are also needed to elucidate other possible biological mechanisms.

The principal function of SHBG has traditionally been that as a transport protein for sex steroids it can regulate circulating concentrations of free (unbound) hormones and their transport to target tissues. However, recent studies have suggested that SHBG might influence the risk of MetS via (1) mediation of steroid hormone signal transduction at the plasma membrane (2) production of a direct effect in endothelial cells through the SHBG receptor and (3) direct effects on the cell by binding to its receptor, acting as a hormone^12, 16, 30^. In addition to early reports of the association between genetic polymorphisms of the SHBG gene and the risk of type 2 diabetes^13, 14^, Pan *et al*. and Xita *et al*. also found that genetic polymorphisms of the SHBG gene were significantly associated with the risk of MetS^16, 31^. Recently, Wang *et al*. used Mendelian randomization that utilizes genetic variants or polymorphisms as proxies for exposures, to evaluate the potential causality of SHBG on the metabolic measures and insulin resistance in three Finnish population-based cohorts. They also found that SHBG is strongly associated with multiple circulating lipids and metabolites, and prospectively associated with the development of insulin resistance and DM even after adjustment for baseline T levels^32^. Those results suggest a causal role for SHBG in the risk of MetS. Further studies are needed to elucidate whether SHBG is merely a biomarker of MetS or could play an active role in the development and progression of MetS.

There are some limitations to this study. First, our design is a cross-sectional study, so we cannot establish cause and effect relationships, but only investigate associations. Second, our results are based on single measurements of serum T levels. Although we have attempted to minimize natural diurnal variation in T levels by always drawing blood samples between 8:00 and 11:00 AM on the day of the screening, the results from single measurement may not accurately represent real serum T levels of study subjects.

In conclusion, the results from a large sample of middle- to old-aged Taiwanese men reveal that subjects with lower serum TT or SHBG levels, but not FT levels are exposed to a higher risk for MetS. In addition, SHBG served as a major predictor for the risk of MetS and was correlated with serum adiponectin and leptin levels that are independent of T. Further studies are needed to elucidate the real role of SHBG in the pathogenesis of MetS and possible mechanisms associated with serum adiponectin and leptin levels.

## Materials and Methods

### Subjects and study protocol

The cross-sectional data of Taiwanese men older than 40 years were collected from a free health screening held between August 2010 and February 2012 by a medical center in Kaohsiung City, Taiwan. Subject selection has been described in detail previously^33, 34^. Ethics approval following the Declaration of Helsinki was authorized by the Institutional Research Ethics Committee of Kaohsiung Medical University Hospital and informed written consent was obtained from each participant before their participation. Exclusion criteria included: (1) subjects who had current malignancy, advanced liver and/or renal disease, major psychiatric disorder, or substance abuse disorders; (2) subjects who were using hormones, anti-androgen treatment, antifungal drugs, or steroidal agents; (3) subjects who had incomplete evaluation^33–35^.

Each participant was interviewed by trained researchers using a structured questionnaire to collect detailed medical, surgical and psychosexual history, current medications and lifestyle data^33, 34^. Subjects were classified as alcohol drinkers or cigarette smokers if they had regularly consumed any alcoholic beverage ≥1 times per week, or had smoked ≥10 cigarettes per week respectively, for at least 6 months. Current users were those who were still using any of these substances within one year before the interview^33, 34^. A habit of regular exercise was defined as participants having exercised aerobically for a minimum of 20 min and perspired, performing this activity regularly more than 1 time per week within one year before the interview^36^.

### Physical Measurements

Physical measurements, including blood pressure, body weight (kg), height (cm) and waist circumference (WC) were recorded for each participant. Blood pressures were measured twice after resting for > 15 min by well‑trained nurses using a mercury sphygmomanometer, and the average values were included in analyses. WC was measured at the midpoint between the inferior costal margin and the superior border of the iliac crest on the mid-axillary line. Body mass index (BMI (kg/m^2^)) was calculated as body weight divided by the square of body height.

### Biochemical and hormone analyses

After more than 8 hours fasting overnight, all participants had 20 cc blood samples drawn between 8:00 and 11:00 AM on the day of the screening for analyses of serum glucose, lipid panels, routine biochemical profiles, adipocytokines, and hormone levels. All analytical methods have been described in detail previously^33, 34^. Briefly, serum adiponectin and leptin were measured using Millipore’s RIA kits (Missouri, USA; Intra-assay coefficients of variation (CV): 3.4%~8.3% and 1.78%~6.21% respectively; Inter-assay CV: 3.0%~6.2% and 6.90%~9.25% respectively)^33^. TT and SHBG levels were determined using a DPC Immulite analyzer (Diamond Diagnostics, Holliston, MA; Inter-assay CV of 8.4% and 4.8% respectively; Intra-assay CV of 5.2% and 3.5% respectively). Free testosterone (FT) level was calculated from serum TT, SHBG, and albumin levels according to the Vermeulen formula^34, 35, 37^.

### Definition of Metabolic syndrome (MetS)

An individual was diagnosed with MetS if he was positive for at least three of the five following criteria: (1) WC ≥ 90 cm; (2) high density lipoprotein (HDL) cholesterol < 40 mg/dL or lipid medication use; (3) triglyceride (TG) ≥ 150 mg/dL or use of lipid‑lowering therapy; (4) blood pressure (BP) ≥ 130 /85 mm Hg or use of antihypertensive medication; (5) fasting blood glucose (FBG) ≥ 100 mg/dL or diagnosed as type 2 DM, in accordance with the modified criteria from the National Cholesterol Treatment Adult Treatment Panel III (NCEP ATP-III) definition proposed by the Bureau of Health Promotion in Taiwan^33, 38^.

### Statistical analysis

Quantitative data have been represented as mean ± standard deviation (SD) and categorical data have been represented by number (n) and percentage. To quantify the differences between subjects with and without MetS, qualitative variables were compared using the chi-square test and Fisher’s exact test, while quantitative variables were compared using the Student’s *t*-test. Correlations between biochemical variables and individual MetS components were analyzed by Spearman’s correlation. Any variables significantly associated with the risk of MetS in the initial analyses were further examined in multivariate regression analyses. Serum T and SHBG levels were examined as quartiles based on the entire population to determine the independent risk of MetS and their association with serum adipocytokine levels. Serum adipocytokine levels were log-transformed to be normally distributed before analyses. To examine the robustness of our analyses, sensitivity test were also performed to exclude subjects with DM in multivariate regression analyses. SPSS version 18.0 (SPSS Inc., Chicago, IL, USA) was used for all statistical analyses.
